# Prospectus of the Chicago Medical Journal for 1860

**Published:** 1859-11

**Authors:** 


PROSPECTUS OF THE CHICAGO MEDICAL JOURNAL FOR 1860.

This Journal will be continued in its present size and form. It will embrace—

1. *Original Articles*, of value; communications from former contributors, and others are earnestly solicited.
2. *Book Notices*, embracing such mention of all important works as will enable our readers to form a correct estimate of the value of each.
3. *Selections* of interest from American and foreign journals.
4. *Editorial* on subjects demanding notice.
5. *Miscellaneous Medical Intelligence.*

It will be the object of the editors to make the Journal valuable as a scientific and practical work, rather than a medium for discussion of medical politics.

We must here invite the attention of our subscribers to the importance of remitting our dues promptly. While a large number have already complied with our terms, there are, we regret to say, still many, well known to us as honorable and responsible gentlemen, who remain indebted to the Journal for one or two year's subscription. We respectfully and urgently solicit of these their immediate attention to this subject.

*Terms*, $2 per annum, payable in advance; if not paid within three months from the commencement of the year (April 1, 1860), the price will be $3.

